# Transcranial direct current stimulation (tDCS) effects on traumatic brain injury (TBI) recovery: A systematic review

**DOI:** 10.1590/1980-57642018dn13-020005

**Published:** 2019

**Authors:** Ana Luiza Zaninotto, Mirret M. El-Hagrassy, Jordan R. Green, Maíra Babo, Vanessa Maria Paglioni, Glaucia Guerra Benute, Wellingson Silva Paiva

**Affiliations:** 1 Speech and Feeding Disorders Lab, MGH Institute of Health Professions (MGH IHP), Boston, USA.; 2 Neuromodulation Center, Spaulding Rehabilitation Hospital, Harvard Medical School (HMS), Boston, USA.; 3 Hospital das Clínicas, Faculdade de Medicina da Universidade de São Paulo, Department of Neurology, São Paulo, SP, Brazil.; 4 Coordinator of the Psychology Course, Centro Universitário São Camilo, SP, Brazil.

**Keywords:** traumatic brain injury, neuronal plasticity, rehabilitation, non-invasive brain stimulation, transcranial direct current stimulation, traumatismo cranioencefálico, plasticidade neuronal, reabilitação, estimulação cerebral não invasiva, estimulação transcraniana por corrente contínua

## Abstract

Traumatic brain injury (TBI) is a major cause of chronic disability. Less than a quarter of moderate and severe TBI patients improved in their cognition within 5 years. Non-invasive brain stimulation, including transcranial direct current stimulation (tDCS), may help neurorehabilitation by boosting adaptive neuroplasticity and reducing pathological sequelae following TBI.

**Methods:**

we searched MEDLINE/PubMed and Web of Science databases. We used Jadad scale to assess methodological assumptions.

**Results:**

the 14 papers included reported different study designs; 2 studies were open-label, 9 were crossover randomized clinical trials (RCTs), and 3 were parallel group RCTs. Most studies used anodal tDCS of the left dorsolateral prefrontal cortex, but montages and stimulation parameters varied. Multiple studies showed improved coma recovery scales in disorders of consciousness, and improved cognition on neuropsychological assessments. Some studies showed changes in neurophysiologic measures (electroencephalography (EEG) and transcranial magnetic stimulation (TMS), correlating with clinical findings. The main methodological biases were lack of blinding and randomization reports.

**Conclusion:**

tDCS is a safe, non-invasive neuromodulatory technique that can be given as monotherapy but may be best combined with other therapeutic strategies (such as cognitive rehabilitation and physical therapy) to further improve clinical cognitive and motor outcomes. EEG and TMS may help guide research due to their roles as biomarkers for neuroplasticity.

## RESUMO.

A lesão cerebral traumática (TCE) é uma das principais causas de incapacidade crônica. Menos de um quarto dos pacientes com TCE moderada e grave melhoraram sua cognição dentro de cinco anos. A estimulação cerebral não invasiva, incluindo a estimulação transcraniana por corrente contínua (ETCC), pode ajudar na reabilitação neurológica, aumentando a neuroplasticidade adaptativa e reduzindo as sequelas patológicas após o TCE.

**Métodos:**

pesquisamos os bancos de dados MEDLINE / PubMed e Web of Science. Usamos a escala de Jadad para avaliar os métodos utilizados nos ensaios clínicos.

**Resultados:**

os 14 artigos incluídos relataram diferentes desenhos de estudo; 2 estudos foram abertos, 9 foram ensaios clínicos randomizados (ECRs) cruzados e 3 foram ECR de grupos paralelos. A maioria dos estudos utilizou a ETCC anódica do córtex pré-frontal dorsolateral esquerdo, mas os parâmetros de montagem e estimulação variaram. Múltiplos estudos mostraram melhoras nas escalas de recuperação de coma em pacientes com distúrbios da consciência e melhora da cognição. Alguns estudos mostraram alterações nas medidas neurofisiológicas (eletroencefalografia (EEG) e estimulação magnética transcraniana (EMT)), correlacionando com os achados clínicos. Os principais vieses metodológicos foram a falta de relatos de cegamento e randomização.

**Conclusão:**

a ETCC é uma técnica neuromodulatória segura e não invasiva que pode ser administrada em monoterapia, mas a utilização da ETCC parece impulsionar os resultados clínicos quando combinada com outras estratégias terapêuticas (como reabilitação cognitiva e fisioterapia). O EEG e o EMT podem ajudar a orientar a pesquisa e tambem mensurar os ganhos clínicos por serem potenciais biomarcadores da neuroplasticidade.

Traumatic brain injury (TBI) is a major cause of death and chronic disability in industrialized^1^ and developing countries,^2^ particularly for young and elderly patients. TBI can lead to transient or permanent physical, cognitive, affective and/or behavioral deficits. Even mild TBI may cause long-term sequalae such as post-concussion syndrome,^3^ potentially leading to neurological disorders and neurodegeneration.^4^^,^ 5 Memory loss is one of the most common deficits following TBI,^6^^-^ 12 and cognitive impairment can be persistent, especially after moderate and severe injury,^12^^-^ 16 resulting in lower functionality and quality of life.^17^^,^ 18 Only 23.7% of moderate and severe TBI patients (older than 16 years) that received inpatient rehabilitation improved in their cognition within 5 years according to the TBI Model Systems National Database, while 24% of the sample reported cognitive decline.^19^ Considering its high disease burden and the limited evidence of cognitive rehabilitation’s effectiveness in TBI,^20^ there is a great need for new and improved therapeutic strategies.

Neuromodulation, such as non-invasive brain stimulation (NIBS) techniques, promotes adaptive neuroplasticity and may prevent or reduce pathological sequela following TBI.^21^^,^ 22 NIBS techniques may improve clinical recovery by facilitating functional and structural neuronal changes, by synaptic strengthening, and by increasing dendritic spines and their connections.^23^^,^ 24 NIBS techniques may potentially improve clinical outcomes beyond conventional rehabilitation and help patients who do not respond to typical therapies.^25^ Transcranial direct current stimulation (tDCS) is a safe NIBS technique studied in various disorders, including TBI.^22^ It involves the application of a low intensity electric current (usually 1 to 2 mA) often using two electrodes placed over the head to modulate cortical activity.^26^ TDCS alters neuronal resting membrane potentials, thereby raising the likelihood of depolarization and increased underlying cortical excitability, or of hyperpolarization and decreased cortical excitability.^24^^,^ 26 Anodal and cathodal tDCS are typically used to increase and decrease excitability respectively, and depending on the montage and stimulation parameters, tDCS can target different cerebral networks, including those involving cognition and motor activity.^27^^,^ 28 As tDCS is relatively safe and cost-effective (24) with only transient adverse effects,^29^^,^ 30 we aimed to systematically review its utility to improve TBI recovery.

The rationale of this systematic review is that TBI is a complex disorder with limited therapeutic options, and that tDCS may be a potential adjuvant neurorehabilitation tool to improve clinical outcomes (e.g., cognitive, motor, and level of consciousness) in TBI. Our hypothesis is that tDCS may improve clinical and surrogate outcomes in TBI, depending on stimulation parameters. Our objective is to answer the following PICOS-based research question: does tDCS improve clinical or surrogate outcomes in adult TBI patients in clinical trials?

## METHODS

Our initial online literature search was performed on MEDLINE/PubMed and Web of Science databases. On Pubmed we used the following MeSH terms: ((traumatic brain injury[MeSH Terms]) OR (tbi[MeSH Terms])) AND ((tDCS[MeSH Terms]) OR (Transcranial Direct Current Stimulation[MeSH Terms]) OR (tDCS[MeSH Terms])). We filtered by date (from 1/1/1900 to 9/15/2018), Species (Human), and Languages (English). On 9/17/2018 we searched Web of Science for the following search string and filters (TS means Topic): (TS=(traumatic brain injury OR tbi)); timespan: 1900-2018; indexes: SCI-EXPANDED, SSCI, A&HCI, CPCI-S, CPCI-SSH, BKCI-S, BKCI-SSH, ESCI, CCR-EXPANDED, IC. We included experimental clinical trials, open label studies and case reports.

Two independent researchers (AZ and MM) reviewed the titles and abstracts. Eligible studies fulfilled the following criteria: experimental studies on adult TBI patients who received tDCS for therapeutic purposes with the primary or exploratory aim of assessing clinical outcomes (e.g., cognitive, motor, or level of consciousness) or surrogate outcomes (e.g., electroencephalogram (EEG), transcranial magnetic stimulation (TMS)), over any duration of time compared to a pre-treatment baseline. We excluded studies that did not meet these criteria, screening first by title, then abstract, then by full text.

We assessed studies for biases by evaluating funding sources. We used the Jadad score to assess publications based on the quality and reporting of the following methods: randomization (0,1, or 2 score); blinding (0,1, or 2 score); and patient flow (0 or 1 score); scores range from 0 to 5 and the higher the score the better the publication.^31^ There was generally no need to contact study authors as the necessary data was available, although we did contact one author to clarify blinding methods.^32^ Our methods follow PRISMA guidelines.

## RESULTS

**Figure 1:**
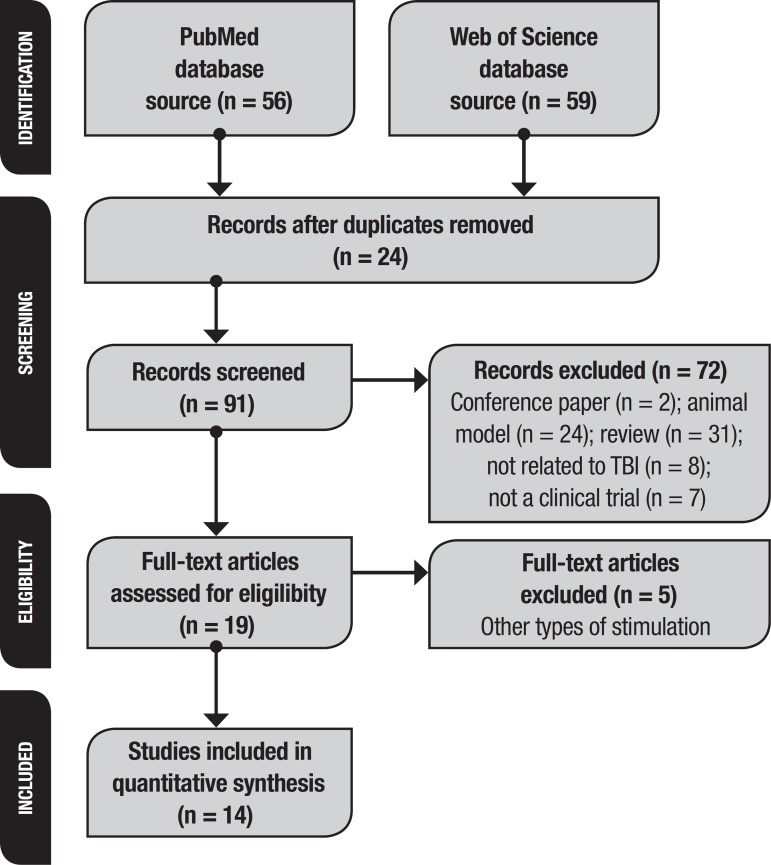
Flow diagram following Prisma Statement.

Of 115 search results (56 from Pubmed, 59 from Web of Science), we found 14 studies that used tDCS in TBI patients and fulfilled our eligibility criteria (Figure 1).

The 14 papers included in our review reported different study designs; 2 studies were open-label case-series^33^^,^ 34 and the rest were double-blind randomized clinical trials (RCTs) - 9 crossover RCTs^32^^,^ 35 ^-^ 42 one of which was semi-randomized,^41^ and 3 parallel group RCTs.^43^^-^ 45 Sample sizes were small, ranging from 5 to 55 participants, and often included other disorders (e.g., anoxia) or healthy controls in addition to TBI. A summary of the 14 papers is presented in Table 1.

**Table 1:** Characteristics of studies using tDCS in patients with TBI.

Author, year	Study design	Total sample size (N)	Electrode placement/polarity	Sponge size	Additional therapies	Stimulation parameters	Total number of tDCS sessions	Primary outcome(s)	Results	Jadad score
Kang etal. 2012	Double-blind, randomized, crossover	N=9	F3 anodal; right supraorbital (Fp2) cathodal	5x5 cm	None	20 min of anodal tDCS, 2 mA; or sham tDCS; 48-hour washout	2	Attention by computerized contrast reaction time task	No significative improvement on RT after tDCS	3
Lesniak et al. 2013	Double-blind, parallel group, pilot study	N=26	F3 anodal; right supraorbital (Fp2) cathodal	5x7 cm	Offline cognitive training	10 min of anodal tDCS, 1 mA; or sham tDCS	15	Episodic memory, working memory and attention	Larger effect size after the intervention compared to sham, but no significant difference between groups	4
Angelakis et al. 2014	Open-label, case series, blinded dose scalation	N=10 (DOC due to TBI n=5)	C3 or F3 anodal; right supraorbital (Fp2) cathodal	5x5 cm	None	Week 1 - 20 min of sham tDCS; Week 2-20 min of anodal tDCS, 1 mA; Weeks 3 and 4 - 20 min of anodal tDCS, 2 mA	20	CRS-R	Conflicting results. Some patients improved up to 2 weeks after stimulation. One patient received 10 extra sessions of tDCS after 3 months and showed improvement. Better improvement on patients with MCS compared to PVS	2
Middleton etal. 2014	Open-label, case series pilot study	N= 5 (n=2 TBI)	Ipsilesional anodal at C3 or C4; and contralesional cathodal	5x5 cm	24 sessions of online physical therapy (3 times per week)	15 min of anodal tDCS, 1.5 mA	24	Fugl-Meyer, Purdue Pegboard, Box and Block, Stroke Impact Scale-16	Positive effects on motor performance up to 6 months after intervention	2
Thibaut etal. 2014	Double-blind, randomized, crossover	N=55 (n=32 DOC due to TBI)	F3 anodal; Fp2 cathodal	5x7 cm	None	20 min of anodal tDCS, 2 mA; or sham tDCS; 48-hour washout	2	CRS-R, GOSe	Improvements for MCS patients on CRS-R total scores. No effect of tDCS on any of the CRS-R subscales on VS/ UWS groups	5
Ulam etal. 2015	Double blind, randomized, parallel group	N=26 (subacute TBI)	F3 anodal; Fp2 cathodal	5x5.6 cm	None	20 min of anodal tDCS, 1 mA; or sham tDCS	10	EEG, neuropsychological assessments	Decreased theta with first session; decreased delta and increased alpha after active tDCS. No changes in sham group. Correlation between decreased delta and improved cognitive tasks in active group	3
Naro etal. 2015	Cross-sectional open-label study	N=45 (n= 20 healthy and n=25 DOC due to TBI or anoxia)	SO anodal (between Fp1 and Fp2); Cz cathodal	5x5 cm anode; 5x7 cm reference electrode	None	10 min of anodal tDCS, 1 mA; or sham tDCS	1	CRS-R, MEP, ICF, ICI, and SICI	Significant effects in all physiological measurements in healthy controls after the tDCS, and on ICI and ICF in DOC patients	2
Sacco etal. 2016	Double-blind, randomized, parallel groups	N=32	F3 or F4 anodal (anode on the lesioned hemisphere and cathode on the other hemisphere); bi-montage F3/ F4 anodal in case of equal hemispheric lesion distribution	5x7 cm	Offline cognitive training	20 min of anodal tDCS, 2 mA; or sham tDCS	10 (twice a day)	TEA, BDI-II, RBANS, AES, fMRI	Shorter reaction times and fewer errors compared to baseline; decreased apathy	2
O'Neil- Pirozzi etal. 2017	Double-blind, crossover pilot study	N=8 (n=4 with chronic severe TBI)	F3 (anodal, cathodal or sham); right supraorbital (Fp2) reference electrode	5x7 cm	None	20 min of anodal or cathodal tDCS, 2 mA; or sham tDCS. 48-hours washout	3	EEG alpha power, P300 amplitude and latency, working memory test	Positive effects on working memory after anodal compared to cathodal tDCS in TBI and control group. No EEG changes	2
Wilke etal. 2017	Double-blind, semi-randomized, crossover study	N=39 (n=17 chronic mild TBI)	C3 anodal; right supraorbital (Fp2) cathodal	5x7 cm anode, 10x10cm cathode	None	20 min of anodal tDCS, 1 mA; or sham tDCS. 7-day washout	2	MRS (GABA concentration), TMS, PCS, cognitive assessment	No changes in any outcome	3
Martens etal. 2018	Double-blind, crossover study	N=27 (n=12 DOC due to TBI)	F3 anodal, right supraorbital (Fp2) cathodal	5x7 cm	None	20 min of anodal tDCS, 2 mA; or sham tDCS; 8-week washout	40 (2-4 weeks stimulation and 8 weeks washout)	Adverse events adherence, CRS-R	Safety, feasibility and behavioral effects - increase in CRS-R scores	5
Estraneo etal. 2017	Double-blind, crossover study	N=23 (n=1 VS due to TBI)	F3 anodal, right supraorbital (Fp2) cathodal	5x7 cm	None	20 min of anodal tDCS, 2 mA; or sham tDCS. One-week washout	10 sessions (5 active and 5 sham stimulation)	CRS-R and EEG	No changes on EEG or CRS-R	4
Thibaut etal. 2017	Double-blind, crossover study	N=21 (n=11 out of 16 DOC due to TBI)	F3 anodal, right supraorbital (Fp2) cathodal	5x7 cm	None	20 min of anodal tDCS, 2 mA; or sham tDCS. One-week washout	10 sessions (5 active and 5 sham stimulation)	CRS-R	Positive treatment effect (CRS-R scores) after one week of anodal tDCS compared to sham	5
Bai etal. 2017	Double-blind, crossover study	N=16 (n=4 VS or MSC due to TBI)	F3 anodal, right supraorbital (Fp2) cathodal	5x5 cm	None	20 min of anodal tDCS, 2 mA; or sham tDCS. 3-day washout	2	TMS (MEP) and EEG	Global cerebral excitability increased in early time windows (0-100 and 100-200 ms) for patients with MCS after anodal tDCS	4

AES: Apathy Evolution Scale; BDI-II: Beck Depression Inventory (2nd edition); CRS-R: Coma Recovery Scale-Revised; DLPFC: dorsolateral prefrontal cortex (F3 on left, F4 on right); DOC: disorder of consciousness; EEG: electroencephalography; GABA: gamma-aminobutyric acid; GOSe: Glasgow Outcome Scale extended; ICF: intracortical facilitation; ICI: intracortical facilitation; M1: Primary Motor Cortex (C3 on left, C4 on right); MEP: motor evoked potential; MRS: magnetic resonance spectroscopy; MSC: Minimal State of Consciousness; PCS: post-concussion syndrome; PVS: persistent vegetative state; RBANS: Repeatable Battery for the Assessment of Neuropsychological Status; SICI: short-interval intracortical inhibition; SO: Supraorbital (orbitofrontal; FP1 on left, FP2 on right); PCS: post concussion syndrome; TEA: Test of Everyday Attention; tDCS: transcraniai direct current stimulation; TMS: transcranial magnetic stimulation; UWS: unresponsive wakefulness syndrome; VS: vegetative State.

### Type of outcomes

The papers reported the use of tDCS in patients with TBI to improve clinical outcomes (mainly coma recovery and cognitive outcomes) and/or surrogate outcomes such as neurophysiological markers (electroencephalography (EEG) and transcranial magnetic stimulation (TMS)), magnetic resonance spectroscopy (MRS) and functional magnetic resonance imaging (fMRI).

We found 7 studies that used tDCS to improve responsiveness in patients with disorders of consciousness (DOC) due to TBI and other brain injuries.^33^^,^ 35 ^-^ 39 ^,^ 42 The only strong evidence of tDCS’ effectiveness to improve functionality as measured by Coma Recovery Scale-Revised (CRS-R) came from the same group. All their studies were crossover RCTs using anodal tDCS (2 mA, current density 0.571 A/m^2^ for 20 minutes) over the left dorsolateral prefrontal cortex (DLPFC) with a right frontopolar reference electrode.^35^^,^ 37 ^,^ 38

A study using TMS-EEG (n=4/16 subjects had TBI) reported global excitability increases early on for MCS patients after anodal left DLPFC tDCS;^39^ the authors described significantly increased global mean field amplitudes of the TMS-evoked potentials with 200 ms of the TMS pulse in MCS patients overall, as opposed to VS patients who also had an increase at up to 100 ms, but a decrease at 300-400 ms. Estraneo et al.^36^ had no positive overall results following left DLPFC anodal tDCS (2 mA over 5 days) on functional (CRS-R) and surrogate (EEG) outcomes. However, this crossover RCT was more heterogeneous than the previous one,^37^ with a mix of subacute and chronic DOC patients.

Six studies used tDCS to improve cognition,^33^^,^ 40 ^,^ 41 ^,^ 43 ^-^ 45 four of which showed no differences between outcomes pre and post-intervention.^40^^,^ 41 ^,^ 43 ^,^ 45 Two parallel-group sham-control RCTs used offline cognitive training after tDCS.^44^^,^ 45 The authors suggested that the combined intervention (tDCS + cognitive training) decreased abnormal hyperactivation, measured by fMRI, often seen in TBI patients.^44^ As to the other studies evaluating cognition, one crossover study^40^ found no improvements in RT using the same parameters as those used successfully by Sacco and colleagues^44^ and at a higher current density (due to smaller electrodes). However, they used only 2 sessions, a right orbitofrontal cathode, had no cognitive training and had a small sample size (n=9), which possibly underpowered the results.

Two studies compared cognitive outcomes to EEG outcomes following left DLPFC tDCS: one parallel-group RCT on subacute TBI reported resting EEG power improvements after one anodal tDCS session (transiently decreased theta slowing at F3), at the end of 10 sessions and the following day (decreased delta plus increased alpha at both F3 and Fp2). It is important to note that this increased normalization (increased physiologic alpha, decreased pathologic delta) occurred under both the Fp2 “cathode” and the F3 “anode”. Additionally, the decreased delta power correlated with improved visual accuracy, color word interference, and brief visual memory task.^43^ Meanwhile, the second study was an exploratory crossover RCT, a single session of anodal tDCS increased word recall in both the TBI and control groups; it also increased P300 amplitude (for oddball task performance) in TBI patients. There was no effect on EEG theta or alpha power.^33^

A semi-randomized crossover study hypothesized that TBI patients would have worse cognition and higher GABA concentration and receptor activity than healthy controls, and that anodal left M1 tDCS would help ameliorate these findings. However, they found no changes in cognition, post-concussion syndrome, TMS or magnetic resonance spectroscopy measures,^41^ possibly due to tDCS response variability or variability in their methodology compared to previous studies.

Finally, only one exploratory open-label study analyzed the effects of online tDCS on motor outcomes in patients with stroke and/or TBI.^34^ This study aimed to assess the feasibility and effectiveness of tDCS sessions. The results showed improvements up to 6 months after the intervention, but the interpretation was based on the effect size, which is not standard considering the small sample.^34^ The TBI patients had mixed results.

**Methodological scores for clinical trials:** We analyzed the quality of the studies using the Jadad score.^31^ Only 3 papers scored 5 out of 5, having clearly reported the randomization and blinding methods as well as dropouts and withdrawals. Most other studies did not report the method used for randomization, or they were open label studies and scored zero for blinding.

## DISCUSSION

When the brain is injured by trauma or other insults, it attempts to ameliorate the deficits resulting from its injury by forming new cortical and subcortical connections and by reorganizing neural networks. However, these compensatory mechanisms are often suboptimal, unable to fully restore function, and may lead to maladaptive effects and further complications such as cognitive impairment. Non-invasive brain stimulation (NIBS) techniques aim to utilize these neuroplastic mechanisms in ways that might target important functions and thereby improve clinical outcomes and quality of life. In other words, NIBS techniques such as tDCS aim to counteract maladaptive neuroplasticity and promote adaptive changes. The search for efficacious adjuvant therapies to improve outcomes in TBI is critical because rehabilitation techniques, and particularly cognitive rehabilitation, often do not lead to complete recovery.

This review aimed to investigate the question: does tDCS improve clinical or surrogate outcomes in adult TBI patients in clinical trials? Most studies showed evidence of positive outcomes (surrogate and/or clinical) in TBI patients after tDCS^32^^-^ 35 ^,^ 37 ^-^ 39 ^,^ 42 ^,^ 44 ^,^ 45 albeit with some methodological variability. Limitations due to heterogeneous procedures are common to rehabilitation studies because the need for tailored therapy makes clinical trial design particularly challenging. Cognition can be especially difficult to target; the exact networks involved in cognitive performance are less clearly delineated than in motor function and are therefore are difficult to target with conventional rehabilitation techniques or with adjuvant therapies such as tDCS. Yet, cognitive problems are a major cause of diminished independence and quality of life in TBI patients,^46^ and they often coincide with - and are confounded by - behavioral and emotional deficits. Any hope for improvement is thus worth investigating. Motor outcomes are also important and merit further investigation in TBI.

Overall the clinical and neurophysiologic results of this systematic review are preliminarily encouraging with regard to coma recovery, cognitive functions and motor recovery in TBI patients. However, further studies are needed to elicit the effects of tDCS parameters, including electrode placement, current density, stimulation duration and interval, as well as its effect on concomitant therapies (and vice versa). Additionally, further studies could help better identify potential tDCS protocol responders based on baseline characteristics.

Considering the risks of polypharmacy in TBI, the potential of tDCS to reduce the need for - and perhaps to counteract the cognitive side effects of - some medications might be very useful. Combining tDCS with cognitive and/or physical training may enhance long-term potentiation (LTP)-like plasticity in the desired region beyond either treatment alone;^47^ however, it is important to understand how to use each of tDCS and other therapies to induce neurophysiologic effects individually before their combined effects can be delineated. This is important to avoid reaching a ceiling effect, which is probably what happened in one study.^45^ It may also be possible to obtain synergy by combining tDCS with another treatment, or to use each treatment to target different functions; conversely, targeting the wrong or opposing networks may cancel the therapeutic effects of each treatment.

Improved biomarkers of neural damage due to TBI may help us better understand the mechanisms underlying tDCS and/or other therapies’ neurophysiologic effects and may also help clinicians predict their clinical effects and monitor therapy. Our review reveals how EEG and TMS markers preliminarily showed changes in some cases, which did - or did not - correlate to clinical outcomes. TBI is a heterogeneous disorder and anything that helps clinicians eventually tailor therapy or identify responders would be helpful, particularly considering the multiple comorbidities and different types of therapy TBI patients may receive.

TMS can be used as NIBS to promote neuroplasticity when used in a repetitive way (rTMS) or as a biomarker to evaluate the integrity of the corticospinal tract. In our review, the TMS cortical silent period was used to investigate the GABAergic pathway in patients with mild TBI,^41^ and to evaluate DLPFC excitability in patients with disorders of consciousness.^39^ While such surrogate markers have limited generalizability to clinical applications, these measures are becoming increasingly correlated over the years. One example is a study published in 2015, in which the authors found a specific TMS threshold with reliable sensitivity to diagnose early stage amyotrophic lateral sclerosis (ALS).^48^

EEG is the other main biomarker used in our review to assess cortical activity after TBI. EEG is clinically used in TBI (especially when severe), in patients admitted to the intensive care unit, to rule out subclinical seizures, to monitor drug effects, and for other clinical purposes.^49^^,^ 50 However, it is not typically used in outpatient settings if there is no history suggestive of seizures. Yet, EEG can be used to follow clinical changes in patients over time even in the presence of medications, as the effects of certain neurological and psychiatric drugs on EEG (e.g., benzodiazepines, etc.) are known. In the context of our review, generalized slowing on EEG is consistent with encephalopathy (if the patient is not sleeping), while pathological focal slowing (especially in the delta range, but also often in the theta range) indicates dysfunction consistent with focal cortical lesions (e.g., stroke, subdural hematoma, abscess, neoplasm, etc.).^51^ Both generalized and focal slowing can variably be seen in TBI patients. Therefore, any decrease in pathological focal slowing is consistent with potentially improved cortical function; for example, in our systematic review, decreased delta power under the electrodes after active tDCS correlated with improved cognitive task performance. Decreased generalized slowing would indicate less or resolved encephalopathy. Eventually, a combination of clinical evaluations, EEG, TMS and/or other neurophysiologic assessments may aid in the development of higher quality tDCS studies in TBI. TMS may be particularly helpful to monitor motor responses.

Overall, the effects of tDCS on clinical outcomes and neurophysiologic markers such as EEG and TMS in TBI patients need to be elucidated in future studies. These studies are worthwhile as heterogeneous disorders require tailored therapy, and tDCS lends itself well to tailoring and individualization based on patient need.

In conclusion, TBI is an unfortunate phenomenon with frequently devastating and heterogeneous clinical outcomes. Cognitive outcomes in TBI are a major source of disability, and few therapeutic options are available. TDCS is a safe, non-invasive neuromodulatory technique that can be given alone (e.g., in comatose patients) but may be best combined with other therapeutic strategies (such as cognitive rehabilitation and physical therapy) to further improve clinical cognitive and motor outcomes. The desired outcomes will have a major impact on networks to target and thus tDCS stimulation parameters and concomitant therapies. The challenges of designing trials for heterogeneous TBI patients necessitate further development of neurophysiologic markers such as EEG and TMS to help track therapeutic progress and guide research.
